# Correction to “Beyond Early Motor Response: Longitudinal Cognitive and Gait Assessments After Extended Lumbar Drainage in Normal Pressure Hydrocephalus”

**DOI:** 10.1111/ene.70204

**Published:** 2025-05-14

Caneva S, Hamedani M, Pesaresi A, et al. Beyond early motor response: Longitudinal cognitive and gait assessments after extended lumbar drainage in normal pressure hydrocephalus. *Eur J Neurol*. 2025; 32:e16567. doi:10.1111/ene.16567

In the above article, there are a couple of errors in the references. In the Method section, page 2, bottom right column, the MMSE is incorrectly referenced to the MoCA test.

The MMSE citation should be inserted as reference [17]: Folstein MF, Folstein SE, McHugh PR. “Mini‐mental state.” A practical method for grading the cognitive state of patients for the clinician. *J Psychiatr Res*. 1975; 12(3): 189–198. doi:10.1016/0022‐3956(75)90026‐6

All subsequent references in the reference list should be renumbered. This updates the reference list from [29] to [30]. The in‐text citation in the last paragraph currently citing [29] should now cite [30].

We apologize for this error.

